# Osteoimmunology in Bone Regeneration

**DOI:** 10.1155/2020/6297356

**Published:** 2020-07-17

**Authors:** Ziqing Li, Puyi Sheng, Chaohong Li, Gongsheng Yuan, Yilun Deng

**Affiliations:** ^1^Department of Anatomy and Cell Biology, School of Dental Medicine, University of Pennsylvania, Philadelphia, USA; ^2^Department of Basic and Translational Sciences, School of Dental Medicine, University of Pennsylvania, Philadelphia, USA; ^3^Department of Joint Surgery, The First Affiliated Hospital of Sun Yat-sen University, Sun Yat-sen University, Guangzhou, China; ^4^Department of Histology and Embryology, Zhongshan School of Medicine, Sun Yat-sen University, Guangzhou, China; ^5^Department of Physiology and Pathophysiology, School of Basic Medical Sciences, Fudan University, Shanghai, China; ^6^Department of Medicine, Hematology & Oncology Division, University of Texas Health San Antonio, San Antonio, USA

Bone has the unique capacity to heal bony injuries without fibrous scar formation via its regeneration process and therefore maintains physiological and mechanical characteristics. This continuous and well-orchestrated regeneration process is also critical for the upkeep of calcium homeostasis throughout one's lifetime [1, 2]. Stimulating bone regeneration is the central aim in orthopedic surgery and in oral and maxillofacial surgery, in order to overcome large bone defects and to cure metabolic/inflammatory bone diseases. Although tissue-engineered materials or artificial bone can help to retain bone stability, biocompatibility and bioresorbability are still significant obstacles for biomaterials to achieve so as to sync with the natural regeneration process. During recent decades, advances in this field have begun to reveal various regulatory molecules shared by the immune system and skeletal system, which depict interesting crosstalk of signaling transducers between immune cells and bone cells [3, 4]. Therefore, osteoimmunology has developed as an essential interdisciplinary field underlying major discoveries concerning bone regeneration and developing targeted therapies for bone diseases. This special issue is aimed at presenting recent research efforts in the crosstalk between the immune system and the skeletal system and their potential application for bone regeneration.

The study by X. Zhang et al. reports the osteoimmunomodulatory properties of microscale magnesium (Mg ions) in stimulating osteogenesis via the immunomodulation between bone marrow stem cells (BMSCs) and macrophages. The authors found that microscale Mg ions could induce M2 phenotype changes of macrophages and inhibited the TLR-NF-*κ*B signaling pathway by the release of anti-inflammatory cytokines. Meanwhile, microscale Mg ions stimulated the expression of osteoinductive molecules in macrophages and promoted osteogenesis of BMSCs through the BMP/SMAD signaling pathway. The results indicated that manipulating Mg ion concentration to a proper microscale can endow the Mg biomaterial with favorable osteoimmunomodulatory properties, thereby providing crucial evidence for improving and modifying the effect of Mg-based bone biomaterials.

The study by C. Fu et al. identifies the novel function of phosphatase and tensin homolog deleted on chromosome 10 (PTEN) as a critical factor in periodontitis and bone remodeling. The authors showed that PTEN decreased in a ligature-induced mouse periodontitis model and was associated with inflammatory factors interleukin 1 (IL-1) and tumor necrosis factor (TNF-*α*) in macrophages. Lack of PTEN activated IL-1 and TNF-*α*, which increased the number of osteoclasts and led to alveolar bone erosion and loss, whereas nanoparticle therapy of PTEN could directly inhibit the inflammatory process and bone erosion in vivo. The study provides a novel insight into understanding the protective effects of PTEN on inflammation and bone remodeling in periodontitis and proposes that PTEN can be used as adjuvant therapy for inflammatory diseases.

The study by J. Yuan et al. investigated the therapeutic effect of genistein on rat temporomandibular joint osteoarthrosis (TMJOA) which was characterized by chronic inflammation and joint cartilage loss. The authors demonstrated that genistein treatment had positive effects on the condylar cartilage renovation, wherein high-dose genistein treatment had better effects on the reversal of OA changes and reduction of the expression of p65 (NF-*κ*B signaling) and inflammatory cytokines (IL-1*β* and TNF-*α*) in male TMJOA rat models. Collectively, the study indicated a better therapeutic effect of high-dose genistein on condyle cartilage damage in TMJOA rats via the suppression of NF-*κ*B expression and inflammatory cytokine activation.

The study by J. Xiong et al. reveals the relationship between dyslipidemia and the risk of osteoarthritis (OA) based on the foundation that autoimmune response affects the homeostasis of the internal environment in the human body and causes self-immune regulation. Through a meta-analysis study comprising 22,501 patients with OA (19,733 with hand OA, 2,679 with knee OA, and 89 with hip OA), the authors stated that OA was higher in those with dyslipidemia compared to those who did not have. Therefore, dyslipidemia might be associated with an increased risk of OA.

The review by H. Wang et al. discusses the current understanding of the varied roles of osteoclasts (OCs) in maintaining skeletal health. The authors summarize the coupling factors that affect the interaction and crosstalk of OCs with osteocytes, mesenchymal stem cells (MSCs), and osteoblasts (OBs), in order to provide a different perspective on recognizing OCs when strategies are created to develop ideal therapeutic agents that target bone remodeling disorders characterized by excessive OC activity.

The study by Z. Chen et al. defines the immune cell landscape of different structures of the knee in OA by using cell-type identification by estimating relative subsets of known RNA transcripts (CIBERSORT) for deconvolution of the global gene expression data. The authors suggested that the immune cell composition in knee OA differed substantially in different anatomical structures of the knee. Meanwhile, activated mast cells were mainly associated with high immune cell infiltration in OA. Moreover, M2 macrophages in the synovium and mast cells in subchondral bone may play essential roles in the pathogenesis of OA.

In conclusion, a comprehensive understanding of ongoing efforts would enable researchers to identify the most efficient approaches in the field and eventually lead to the successful discovery of therapeutics. The guest editorial team wishes that this special issue will help in evidencing researches from multiple disciplines in this area and encourage future collaborations from multidisciplinary aspects.

## References

[1] Martin T. J., Seeman E. (2008). Bone remodelling: its local regulation and the emergence of bone fragility. *Best Practice & Research. Clinical Endocrinology & Metabolism*.

[2] Hadjidakis D. J., Androulakis I. I. (2006). Bone remodeling. *Annals of the New York Academy of Sciences*.

[3] Xiao L., Zhou Y., Friis T., Beagley K., Xiao Y. (2019). S1P-S1PR1 signaling: the "Sphinx" in osteoimmunology. *Frontiers in Immunology*.

[4] Kim B. J., Koh J. M. (2019). Coupling factors involved in preserving bone balance. *Cellular and Molecular Life Sciences*.

